# Diisopropyl­ammonium methane­sulfonate

**DOI:** 10.1107/S1600536811029382

**Published:** 2011-07-30

**Authors:** Guido J. Reiss, Michaela K. Meyer

**Affiliations:** a Institut für Anorganische Chemie und Strukturchemie, Lehrstuhl II: Material- und Strukturforschung, Heinrich-Heine-Universität Düsseldorf, Universitätsstrasse 1, D-40225 Düsseldorf, Germany

## Abstract

The title molecular salt, C_6_H_16_N^+^·CH_3_SO_3_^−^, has been determined at 150 K. Two diisopropyl­ammonium cations (dipH) and two anions form N—H⋯O hydrogen-bonded cyclic dimers lying around centers of symmetry. Only two of the three O atoms of the methane­sulfonate anion are involved in hydrogen bonding, resulting in slightly longer S—O bond lengths. The title structure represents an example of a sulfonate anion that is part of a hydrogen-bonding *R*_4_^4^(12) graph-set motif, which is well known for related dipH acetates. Additionally, the Raman and the IR spectroscopic data for the title compound are presented.

## Related literature

For simple dipH salts, see: Bajorat & Reiss (2007 ▶); Reiss (1998 ▶, 2002 ▶); Reiss (2010*a* ▶,*b* ▶); Reiss & Engel (2004 ▶); Reiss & Meyer (2010 ▶); Sada *et al.* (2004 ▶), Summers *et al.* (1998 ▶). For spectroscopic data for sulfonate salts, see: Thomson (1972 ▶); Genceli Guner *et al.* (2010 ▶). For graph-set analysis, see: Etter *et al.* (1990 ▶).
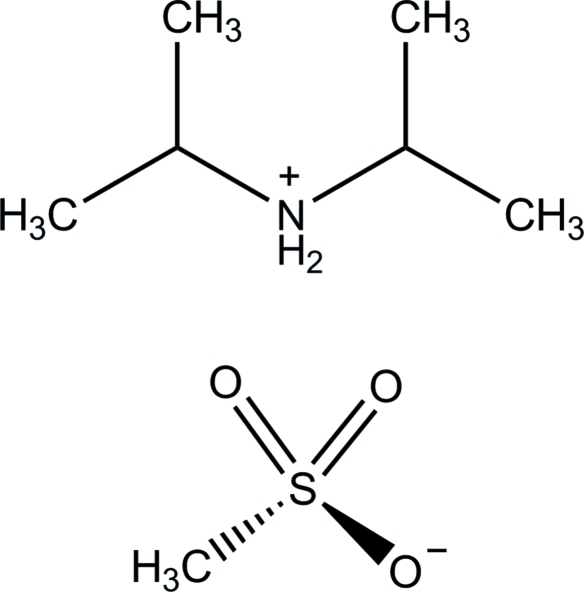


## Experimental

### 

#### Crystal data

C_6_H_16_N^+^·CH_3_SO_3_^−^
*M* *_r_* = 197.29
Monoclinic, 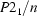

*a* = 8.88154 (13) Å
*b* = 8.53537 (13) Å
*c* = 14.5784 (2) Å
β = 101.8161 (15)°
*V* = 1081.73 (3) Å^3^
*Z* = 4
Mo *K*α radiation
μ = 0.27 mm^−1^
*T* = 150 K
0.65 × 0.25 × 0.10 mm

#### Data collection

Oxford Diffraction Xcalibur Eos diffractometer
Absorption correction: multi-scan (*CrysAlis PRO*; Oxford Diffraction, 2009 ▶) *T*_min_ = 0.951, *T*_max_ = 1.000
25064 measured reflections
3149 independent reflections
2782 reflections with *I* > 2σ(*I*)
*R*_int_ = 0.024

#### Refinement

*R*[*F* ^2^ > 2σ(*F* ^2^)] = 0.027
*wR*(*F* ^2^) = 0.060
*S* = 1.03
3149 reflections
146 parameters
H atoms treated by a mixture of independent and constrained refinement
Δρ_max_ = 0.34 e Å^−3^
Δρ_min_ = −0.32 e Å^−3^

### 

Data collection: *CrysAlis PRO* (Oxford Diffraction, 2009 ▶); cell refinement: *CrysAlis PRO*; data reduction: *CrysAlis PRO*; program(s) used to solve structure: *SHELXS97* (Sheldrick, 2008 ▶); program(s) used to refine structure: *SHELXL97* (Sheldrick, 2008 ▶); molecular graphics: *DIAMOND* (Brandenburg, 2010 ▶); software used to prepare material for publication: *publCIF* (Westrip, 2010 ▶).

## Supplementary Material

Crystal structure: contains datablock(s) I, global. DOI: 10.1107/S1600536811029382/qm2018sup1.cif

Structure factors: contains datablock(s) I. DOI: 10.1107/S1600536811029382/qm2018Isup2.hkl

Supplementary material file. DOI: 10.1107/S1600536811029382/qm2018Isup3.mol

Supplementary material file. DOI: 10.1107/S1600536811029382/qm2018Isup4.cml

Additional supplementary materials:  crystallographic information; 3D view; checkCIF report

## Figures and Tables

**Table 1:** Selected geometric parameters (Å, °)

S1—O2	1.4433 (9)
S1—O1	1.4596 (8)
S1—O3	1.4600 (8)
S1—C1	1.7560 (12)

**Table 2:** Hydrogen-bond geometry (Å, °)

*D*—H⋯*A*	*D*—H	H⋯*A*	*D*⋯*A*	*D*—H⋯*A*
N1—H11⋯O1	0.885 (13)	1.944 (13)	2.8168 (11)	168.5 (12)
N1—H12⋯O3^i^	0.891 (13)	1.919 (13)	2.7944 (11)	166.9 (12)

Symmetry code: (i) 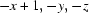.

**Fig. 1.**
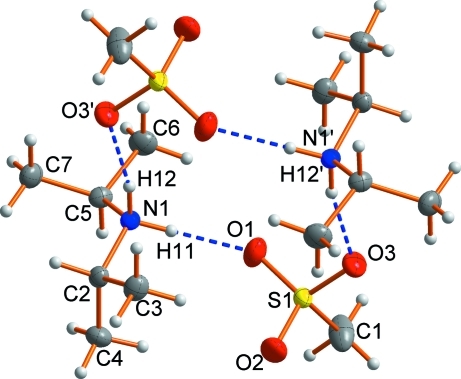
Hydrogen atoms are drawn with an arbitrary radius and the displacement ellipsoids are shown at the 50% probability level; ' = 1 - x,-y,-z.

## supplementary crystallographic information

### Comment

More than one hundred structures are listed to date in the Cambridge Crystallographic Database, which include a diisopropylammonium cation (dipH). In most cases the dipH cation is the counter cation of bigger anionic species *e.g.* anionic complexes and clusters. A limited number of structures are reported that are solely constructed by small classical anions and the dipH cation. In these cases the structure type is strongly dependent on the geometry and the ability of these anions to form hydrogen bonds. The structures reported so far range from a quasi-molecular (dipH)_2_[SiF_6_] (Reiss, 1998), over cyclic dimers for some acetates (*e.g.*: Sada *et al.*, 2004) and *e.g.* a substituted phosphate (Summers *et al.*, 1998), to one-dimensional polymers for the halogenides and some pseudo-halogenides (literature cited in Reiss & Meyer, 2010). Even a two-dimensional hydrogen bonded network for the (dipH)_2_SO_4_ (Reiss & Engel, 2004) and three-dimensional networks for dipH[IrCl_6_] (Reiss, 2002) and (dipH)_2_[CdCl_4_] (Reiss, 2010*a*) have been reported. The asymmetric unit of the title compound, which crystallizes in the centrosymmetric space group *P*2_1_/*n*, consists of one formular unit. The structure is composed of centrosymmetric, hydrogen bonded, cyclic dimers which are composed of two dipH cations and two methanesulfonate anions arranged as rings (Fig. 1). The graph set (Etter *et al.*, 1990) for these hydrogen bonded dimers is *R*_4_^4^(12). All N–C, C–C, S–O, and S–C bond lengths are generally within the expected range. The two S–O bond lengths of the oxygen atoms involved in hydrogen bonding (O1 and O3) are longer than the third one which is in accord to our anticipation (Table 1). The N–H···O hydrogen bonds with donor-acceptor distances close to 2.79–2.82 Å (Table 2) are in the range of medium strong hydrogen bonds. The *R*_4_^4^(12) hydrogen bonding motif is well known for dipH acetates (Reiss & Meyer, 2010, Sada *et al.* 2004). Combinations of the dipH cation with structurally similar anions like [NO_3_]^–^ (Reiss, 2010*b*), [ClO_4_]^-^ (Bajorat & Reiss, 2007) and various substituted carboxylate anions can be described as the generalized synthon: {dipH}^+^{O_2_*XR_y_*}^-^ (*X* = C, Cl, N; *y* = 1,2, *R* = O, C). The title structure is a further example with this supramolecular synthon and the typical cyclic dimer.

### Experimental

Diisopropylammonium methanesulfonate, ((CH_3_)_2_CH)_2_NH_2_[CH_3_SO_3_] was prepared by the reaction of 0.72 g (7.12 mmol) diisopropylamine and 1.48 g (15.40 mmol) methanesulfonic acid at room temperature. From colourless solution small block-shaped crystals were obtained within a few days. A Raman spectrum was measured using a Bruker MULTIRAM spectrometer; Nd:YAG-Laser at 1064 nm; RT-InGaAS-detector; 4000–70 cm^-1^: 3022(sh), 2986(ν(C–H), s), 2944(ν(C–H), *vs*), 2784(ν(N–H), w), 1462 (δ(C–H, m), 1429(δ(C–H, m), 1350(w), 1315(w), 1227(w), 1183(w), 1158(w), 1099 (w), 1044 (ν(S–O),*vs*), 958 (w), 914(w), 808(*s*), 778(ν(C–S), s), 558(δ(S–O), s), 528(*m*), 470(*m*), 392(w), 348(ρ(S–O), br), 83(*m*). IR data collected on a Digilab FT3400 spectrometer using a MIRacle ATR unit (Pike Technologies); 4000–560 cm^-1^: 3019(ν(C–H), s), 2988 (sh), 2941(*m*), 2870(ν(C–H), s), 2781(ν(N–H), m), 2514(*m*), 1625(w), 1601(w), 1483(δ(C–H, m), 1416(w), 1397(*m*), 1331(w), 1314(w), 1206(*vs*), 1177(sh), 1153(ν_s_(S–O), *vs*), 1100(*m*), 1040(ν_a_(S–O), *vs*), 977(w), 954(w), 837(w), 805(w), 776(ν(C–S), s), 551(δ(S–O)s). Band assignments for Raman and IR spectroscopic data were made by comparing results of earlier work done on sodium- and caesium methanesulfonate (Thomson, 1972), magnesium sulfonate (Genceli Guner *et al.*, 2010) and dipHCl (Reiss & Meyer 2010).

### Refinement

A single-crystal suitable for structure determination was harvested from the mother liquor and directly transferred into the cooling stream of an Oxford-Xcalibur diffractometer equipped with an EOS-CCD detector. Data collection was performed at 150 K. All hydrogen atoms were located *via* difference Fourier synthesis. Positional parameters of hydrogen atoms of the NH_2_ and the CH group were refined freely. All hydrogen atoms belonging to methyl groups were refined using a riding model with restrained angles and C–H distances but allowed to rotate about the C–C bond. Anisotrope displacement parameters of all non hydrogen atoms and individual isotropic displacement parameters for all hydrogen atoms were refined freely.

### Crystal data

**Table:**

C_6_H_16_N^+^·CH_3_SO_3_^−^	*F*(000) = 432
*M**_r_* = 197.29	*D*_x_ = 1.211 Mg m^−^^3^
Monoclinic, *P*2_1_/*n*	Mo *K*α radiation, λ = 0.71073 Å
Hall symbol: -P 2yn	Cell parameters from 18202 reflections
*a* = 8.88154 (13) Å	θ = 3.4–32.0°
*b* = 8.53537 (13) Å	µ = 0.27 mm^−^^1^
*c* = 14.5784 (2) Å	*T* = 150 K
β = 101.8161 (15)°	Needle, colourless
*V* = 1081.73 (3) Å^3^	0.65 × 0.25 × 0.10 mm
*Z* = 4	

### Data collection

**Table:**

Oxford Diffraction Xcalibur Eos diffractometer	3149 independent reflections
Radiation source: fine-focus sealed tube	2782 reflections with *I* > 2σ(*I*)
graphite	*R*_int_ = 0.024
Detector resolution: 16.27 pixels mm^-1^	θ_max_ = 30.0°, θ_min_ = 4.1°
ω scans	*h* = −12→12
Absorption correction: multi-scan (*CrysAlis PRO*; Oxford Diffraction, 2009)	*k* = −12→12
*T*_min_ = 0.951, *T*_max_ = 1.000	*l* = −20→20
25064 measured reflections	

### Refinement

**Table:**

Refinement on *F*^2^	Secondary atom site location: difference Fourier map
Least-squares matrix: full	Hydrogen site location: difference Fourier map
*R*[*F*^2^ > 2σ(*F*^2^)] = 0.027	H atoms treated by a mixture of independent and constrained refinement
*wR*(*F*^2^) = 0.060	*w* = 1/[σ^2^(*F*_o_^2^) + (0.01*P*)^2^ + 0.55*P*] where *P* = (*F*_o_^2^ + 2*F*_c_^2^)/3
*S* = 1.03	(Δ/σ)_max_ < 0.001
3149 reflections	Δρ_max_ = 0.34 e Å^−^^3^
146 parameters	Δρ_min_ = −0.32 e Å^−^^3^
0 restraints	Extinction correction: *SHELXL97* (Sheldrick, 2008), Fc^*^=kFc[1+0.001xFc^2^λ^3^/sin(2θ)]^-1/4^
Primary atom site location: structure-invariant direct methods	Extinction coefficient: 0.0276 (9)

### Special details

**Table:**

Experimental. CrysAlisPro, Oxford Diffraction Ltd., Version 1.171.33.52, Empirical absorption correction using spherical harmonics, implemented in SCALE3 ABSPACK.
Geometry. All e.s.d.'s (except the e.s.d. in the dihedral angle between two l.s. planes) are estimated using the full covariance matrix. The cell e.s.d.'s are taken into account individually in the estimation of e.s.d.'s in distances, angles and torsion angles; correlations between e.s.d.'s in cell parameters are only used when they are defined by crystal symmetry. An approximate (isotropic) treatment of cell e.s.d.'s is used for estimating e.s.d.'s involving l.s. planes.
Refinement. Refinement of *F*^2^ against ALL reflections. The weighted *R*-factor *wR* and goodness of fit *S* are based on *F*^2^, conventional *R*-factors *R* are based on *F*, with *F* set to zero for negative *F*^2^. The threshold expression of *F*^2^ > σ(*F*^2^) is used only for calculating *R*-factors(gt) *etc*. and is not relevant to the choice of reflections for refinement. *R*-factors based on *F*^2^ are statistically about twice as large as those based on *F*, and *R*- factors based on ALL data will be even larger.

### Fractional atomic coordinates and isotropic or equivalent isotropic displacement parameters (Å^2^)

**Table:**

	*x*	*y*	*z*	*U*_iso_*/*U*_eq_	
S1	0.38147 (3)	0.02594 (3)	0.180331 (16)	0.01976 (7)	
O1	0.47025 (9)	0.13813 (10)	0.13749 (6)	0.0355 (2)	
O2	0.47472 (10)	−0.09928 (12)	0.22849 (6)	0.0408 (2)	
O3	0.24712 (9)	−0.03058 (10)	0.11340 (5)	0.02989 (17)	
C1	0.30699 (15)	0.12931 (19)	0.26524 (9)	0.0442 (3)	
H1A	0.2477	0.0595	0.2955	0.065 (5)*	
H1B	0.2424	0.2128	0.2357	0.062 (5)*	
H1C	0.3901	0.1721	0.3109	0.066 (5)*	
N1	0.74609 (9)	0.09843 (10)	0.07323 (6)	0.01840 (16)	
H11	0.6562 (15)	0.0976 (15)	0.0906 (9)	0.028 (3)*	
H12	0.7329 (14)	0.0729 (15)	0.0128 (9)	0.028 (3)*	
C2	0.84677 (11)	−0.02552 (12)	0.12848 (7)	0.02104 (18)	
H2	0.9413 (14)	−0.0236 (14)	0.1056 (8)	0.022 (3)*	
C3	0.76690 (13)	−0.18212 (12)	0.10422 (8)	0.0283 (2)	
H3A	0.6709	−0.1827	0.1248	0.036 (4)*	
H3B	0.8313	−0.2649	0.1349	0.039 (4)*	
H3C	0.7480	−0.1977	0.0376	0.037 (4)*	
C4	0.87442 (13)	0.00990 (14)	0.23269 (7)	0.0286 (2)	
H4A	0.9296	0.1069	0.2452	0.036 (4)*	
H4B	0.9337	−0.0731	0.2670	0.037 (4)*	
H4C	0.7775	0.0183	0.2517	0.037 (4)*	
C5	0.80229 (12)	0.26542 (12)	0.08130 (7)	0.02175 (19)	
H5	0.8146 (13)	0.2921 (14)	0.1445 (8)	0.022 (3)*	
C6	0.67707 (14)	0.36632 (13)	0.02325 (8)	0.0322 (2)	
H6A	0.6606	0.3347	−0.0412	0.041 (4)*	
H6B	0.7081	0.4742	0.0286	0.049 (4)*	
H6C	0.5834	0.3538	0.0457	0.041 (4)*	
C7	0.95364 (13)	0.27936 (14)	0.04905 (8)	0.0296 (2)	
H7A	1.0290	0.2129	0.0868	0.041 (4)*	
H7B	0.9884	0.3861	0.0553	0.042 (4)*	
H7C	0.9393	0.2479	−0.0154	0.036 (4)*	

### Atomic displacement parameters (Å^2^)

**Table:**

	*U*^11^	*U*^22^	*U*^33^	*U*^12^	*U*^13^	*U*^23^
S1	0.01698 (10)	0.02503 (12)	0.01773 (10)	0.00188 (9)	0.00463 (8)	−0.00071 (9)
O1	0.0289 (4)	0.0331 (4)	0.0485 (5)	−0.0013 (3)	0.0171 (4)	0.0074 (4)
O2	0.0310 (4)	0.0471 (5)	0.0445 (5)	0.0131 (4)	0.0083 (4)	0.0205 (4)
O3	0.0267 (4)	0.0403 (4)	0.0216 (3)	−0.0034 (3)	0.0022 (3)	−0.0084 (3)
C1	0.0305 (6)	0.0663 (9)	0.0370 (6)	−0.0021 (6)	0.0101 (5)	−0.0286 (7)
N1	0.0188 (4)	0.0194 (4)	0.0171 (4)	−0.0008 (3)	0.0040 (3)	−0.0005 (3)
C2	0.0193 (4)	0.0209 (4)	0.0238 (4)	0.0026 (4)	0.0064 (3)	0.0024 (4)
C3	0.0306 (5)	0.0205 (5)	0.0350 (6)	−0.0003 (4)	0.0096 (4)	0.0007 (4)
C4	0.0295 (5)	0.0322 (6)	0.0223 (5)	0.0020 (4)	0.0009 (4)	0.0043 (4)
C5	0.0269 (5)	0.0187 (4)	0.0196 (4)	−0.0025 (4)	0.0045 (4)	−0.0015 (3)
C6	0.0368 (6)	0.0235 (5)	0.0360 (6)	0.0049 (4)	0.0067 (5)	0.0058 (4)
C7	0.0278 (5)	0.0295 (6)	0.0317 (5)	−0.0080 (4)	0.0066 (4)	0.0030 (4)

### Geometric parameters (Å, °)

**Table:**

S1—O2	1.4433 (9)	C3—H3B	0.9600
S1—O1	1.4596 (8)	C3—H3C	0.9600
S1—O3	1.4600 (8)	C4—H4A	0.9600
S1—C1	1.7560 (12)	C4—H4B	0.9600
C1—H1A	0.9600	C4—H4C	0.9600
C1—H1B	0.9600	C5—C6	1.5196 (15)
C1—H1C	0.9600	C5—C7	1.5169 (14)
N1—C5	1.5068 (13)	C5—H5	0.934 (12)
N1—C2	1.5076 (12)	C6—H6A	0.9600
N1—H11	0.885 (13)	C6—H6B	0.9600
N1—H12	0.891 (13)	C6—H6C	0.9600
C2—C4	1.5189 (14)	C7—H7A	0.9600
C2—C3	1.5206 (14)	C7—H7B	0.9600
C2—H2	0.964 (12)	C7—H7C	0.9600
C3—H3A	0.9600		
			
O2—S1—O1	112.80 (5)	N1—C2—C4	110.58 (8)
O2—S1—O3	112.92 (6)	N1—C2—C3	107.17 (8)
O1—S1—O3	111.71 (5)	C4—C2—C3	112.32 (9)
O2—S1—C1	106.90 (7)	N1—C2—H2	105.4 (7)
O1—S1—C1	106.65 (7)	C4—C2—H2	111.6 (7)
O3—S1—C1	105.24 (5)	C3—C2—H2	109.4 (7)
C5—N1—C2	118.12 (8)	N1—C5—C6	107.44 (8)
C5—N1—H11	106.8 (9)	N1—C5—C7	110.51 (8)
C2—N1—H11	108.2 (8)	C6—C5—C7	112.14 (9)
C5—N1—H12	106.4 (8)	N1—C5—H5	106.1 (8)
C2—N1—H12	107.5 (8)	C6—C5—H5	109.6 (7)
H11—N1—H12	109.6 (11)	C7—C5—H5	110.8 (7)
			
C5—N1—C2—C4	59.81 (11)	C2—N1—C5—C6	−177.19 (8)
C5—N1—C2—C3	−177.47 (8)	C2—N1—C5—C7	60.18 (11)

### Hydrogen-bond geometry (Å, °)

**Table:**

*D*—H···*A*	*D*—H	H···*A*	*D*···*A*	*D*—H···*A*
N1—H11···O1	0.885 (13)	1.944 (13)	2.8168 (11)	168.5 (12)
N1—H12···O3^i^	0.891 (13)	1.919 (13)	2.7944 (11)	166.9 (12)

Symmetry codes: (i) −*x*+1, −*y*, −*z*.
